# Understanding the Mechanism of the Structure-Dependent Mechanical Performance of Carbon-Nanotube-Based Hierarchical Networks from a Deformation Mode Perspective

**DOI:** 10.3390/nano13243119

**Published:** 2023-12-12

**Authors:** Xian Shi, Xiaoqiao He, Xuefeng Liu

**Affiliations:** 1 School of Civil Engineering, Suzhou University of Science and Technology, Suzhou 215009, China; shixian@usts.edu.cn; 2 Department of Architecture and Civil Engineering, City University of Hong Kong, Tat Chee Avenue, Hong Kong; bcxqhe@cityu.edu.hk; 3 Center for Advanced Structural Materials, Shenzhen Research Institute, City University of Hong Kong, Shenzhen 518057, China; 4 College of Hydraulic and Environmental Engineering, China Three Gorges University, Yichang 443002, China; 5 Department of Mechanics and Aerospace Engineering, Southern University of Science and Technology, Shenzhen 518055, China

**Keywords:** CNT network, molecular dynamics simulation, mechanical performance

## Abstract

Carbon nanotube (CNT)-based networks have wide applications, in which structural design and control are important to achieve the desired performance. This paper focuses on the mechanism behind the structure-dependent mechanical performance of a CNT-based hierarchical network, named a super carbon nanotube (SCNT), which can provide valuable guidance for the structural design of CNT-based networks. Through molecular dynamic (MD) simulations, the mechanical properties of the SCNTs were found to be affected by the arrangement, length and chirality of the CNTs. Different CNT arrangements cause variations of up to 15% in the ultimate tensile strains of the SCNTs. The CNT length determines the tangent elastic modulus of the SCNTs at the early stage. Changing the CNT chirality could transform the fracture modes of the SCNT from brittle to ductile. The underlying mechanisms were found to be associated with the deformation mode of the SCNTs. All the SCNTs undergo a top-down hierarchical deformation process from the network-level angle variations to the CNT-level elongations, but some vital details vary, such as the geometrical parameters. The CNT arrangement induces different deformation contributors of the SCNTs. The CNT length affects the beginning point of the CNT elongation deformation. The CNT chirality plays a crucial role in the stability of the junction’s atomic topology, where the crack propagation commences.

## 1. Introduction

Despite being discovered over 30 years ago [1,2], there continues to be a constant stream of progress associated with the carbon nanotube (CNT) because of its excellent mechanical and electronic properties [3,4]. One of the important methods to achieve the practical applications of CNTs is to assemble them into macro-structures, including CNT-based yarns, fibers, films and foams [5,6,7] as well as composite structures such as hetero-junctions and hybrid nanocomposites [8,9]. These CNT-based structures have shown promising potential in various fields, from flexible electronic devices to biological engineering [10,11].

Compared with the random distribution of CNTs, ordered CNT-based structures have prominent advantages for achieving a better performance. When applied in Li–oxygen batteries, the ordered CNT structure benefited the transportation of Li^+^ ions, gas, and electrons [12]. Super-aligned CNTs enhance both the mechanical and electrical properties of copper [13]. Moreover, generating covalent connections between the CNT units is significant for ensuring the excellent performance of CNT-based structures. A three-dimensional CNT sponge with covalent junctions has shown distinct hyperelasticity [14]. The covalent interconnection of the CNT network enables the EP composite to have enhanced thermal conductivity [15]. A review of the covalent three-dimensional networks of graphene and CNTs reported their better adsorption of environmental pollutants and catalytic conversion [16].

Therefore, structure control and assembly are crucial in the perpetration of CNT-based structures. With the rapid advancements in nanotechnology, plenty of breakthroughs have been achieved for the controlled preparation of CNT-based structures. The controlled chirality and shape growth of a super-long CNT has been achieved [17,18] and CNTs have been connected into ‘X’-, ‘Y’- or ‘T’-like covalent junctions [19,20]. Bottom-up approaches have been developed for the fabrication of CNT-based structures, from the template synthetic method [21,22]. Additionally, nanoscale welding and controlling have enabled ordered and multi-scale CNT assemblies [23,24].

When precise control becomes accessible, the structural design of CNT-based structures will be more and more important. In the diversified applications of CNT-based structures, a distinct performance is required to fulfill different functions in various applications [25], which should be guided by the proper design strategy. Through rational structural design, the optimization of performance can be achieved for CNT-based structures and the controlled synthesis of CNT-based structures is more targeted and valuable [26,27].

Theoretical studies can effectively predict the performance of certain materials or structures. Until now, there have been a variety of theoretical studies conducted on CNTs [28,29,30]. Moreover, numerous novel CNT-based structures have been proposed with many attractive features [31,32,33]. Different CNT-based networks are reported to possess superior flexibility, such as the CNT–graphene hybrid structure [34] and the super carbon nanotube (SCNT) [35]. CNT-based super honeycomb structures were also reported to possess tunable thermal conductivity in three dimensions [36] and defect tolerance in the load-transferring process [37].

These studies of CNT-based structures discussed the changing properties induced by variations in the geometry parameters. There are interpretations of the mechanism in these studies, but the corresponding mechanism is simply explained without the explicit illustration of the variations in the structure. As an essential issue in the structural design of CNT-based networks, the underlying mechanism behind the effect of geometry parameters needs to be clarified and further analysis should be carried out.

In this study, we applied molecular dynamic (MD) simulations to investigate the tensile performance of a SWCNT-based hierarchical network, named SCNT. The controllable mechanical properties are presented and the underlying mechanisms of the geometry effect are revealed by analyzing the structure evolution. The structure-dependent mechanical performance of SCNTs was found to relate to the transformation of the deformation mode, which is essentially induced by the hierarchical structure of SCNTs. Furthermore, a structural design strategy was developed for SCNTs that is expected to provide valuable information on the structural design of other CNT-based networks.

## 2. Materials and Methods

### 2.1. Structure of the SCNT

**Figure 1:**
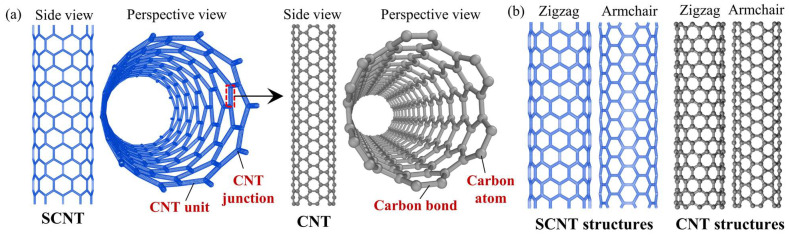
(**a**) Illustrations of the structures of SCNT and CNT; (**b**) Geometrical structures of CNTs and SCNTs with zigzag and armchair chiralities.

The structure of the SCNT is presented in Figure 1a. The SCNT has a self-similar structure to the SWCNT, which is just like an amplified version of the SWCNT with the same tubular overall shape and the honeycomb network geometry. To construct an SCNT, SWCNTs are connected into Y-shaped junctions and then these junctions are assembled into the resulting structures. Based on the topology analysis of the SWCNT connection [38,39], pentagonal and heptagonal carbon rings are introduced at the connecting areas of the junctions. For convenience, the term ‘CNT’ is generally used to represent ‘SWCNT’ in the subsequent paragraphs.

The structures of CNTs vary as the arrangements of carbon bonds and are distinguished by the chirality vector. In terms of chirality, CNT structures can be categorized into three typical types: armchair, zigzag and chiral. Similarly, SCNTs possess various chiralities due to the different arrangements of CNT units. In this study, both zigzag and armchair chirality are applied to both SCNTs and CNTs, as shown in Figure 1b.

However, the structure of the SCNT would also vary as the change in CNT length and topology. Three geometry parameters determine the resulting structures of SCNTs, including CNT chirality, CNT length and CNT arrangement. Therefore, the structure of the SCNT is hierarchical, determined by the structure settings at both the CNT unit level and the network level.

For a better representation of the hierarchical structures of SCNTs, we use the expression of [*N*,*M*]@(*n*,*m*)-*l* to denote different geometry settings of SCNT models. Here, (*n*,*m*) represents the chiral vector of the CNT. The structure organizations at the SCNT level are also identified as an analogous chirality parameter as the CNT, which is [*N*,*M*]. The letter *l* indicates the length of CNT applied to fabricate the SCNT models.

In this study, seven different SCNT models are established. Two classical types of chirality are adopted for CNTs. The (6,6) is the armchair chirality and the (10,0) is the zigzag chirality. For the SCNT, there are three settings of chirality, including [6,6], [3,3] and [10,0]. The first two settings belong to armchair chirality and the last one is the zigzag chirality. The detailed geometry parameters of all SCNT models are presented in Table 1. A detailed illustration of the geometry parameters of both the CNT and the SCNT can be found in Figure S1 of the Supplementary Materials.

### 2.2. Methodology

Selecting a suitable method of simulation is crucial as it needs to balance computational efficiency and reasonable accuracy in obtaining results. Density functional theory (DFT), based on quantum mechanics, can accurately calculate the physical properties and nanoscale morphology of atomic systems, including detailed electron interactions. However, it is only efficient for systems with a few hundred atoms [40]. On the other hand, the classical MD method cannot provide electronic information like DFT, but it can still yield reliable motions and properties of atom/molecule systems by applying empirical potentials derived from quantum mechanics. Additionally, the classical MD method is more effective than DFT when dealing with large-scale systems.

In this study, all SCNT models contain more than one hundred thousand atoms, for which the calculation efficiency is very important. Additionally, the focus of this study is on the mechanical properties and deformation analysis, rather than detailed electronic information. Considering both accuracy and efficiency, the MD method was selected to investigate the tensile performances of the SCNTs. The Adaptive Intermolecular Reactive Empirical Bond Order (AIREBO) potential [41] was employed to describe the interactions between carbon atoms.

The reliability of the AIREBO potential in simulating the mechanical properties and fracture of graphene or CNT can be validated through numerous previous studies. Memarian et al. [42] reported that results within DFT and MD with AIREBO are the most accurate methods regarding the bond length. Liu et al. [43] concluded that the results of Young’s modulus and Cauchy ultimate strength of defect-free graphene obtained from MD simulations with AIREBO potential agree well with the DFT results. According to a review paper [40], AIREBO is considered the most accurate interatomic potential for simulating the mechanical, fracture and thermal properties of single-layered graphene. While some studies reported the inaccuracy of the AIREBO potential, these problems are mostly limited to special conditions such as high temperature and extreme pressure [44,45]. Furthermore, the AIREBO potential has been applied in considerable studies of CNT/graphene-based structures or composites [35,46]. Therefore, the MD method with AIREBO potential was deemed reasonable and acceptable for investigating the SCNT tension in this study.

**Figure 2:**
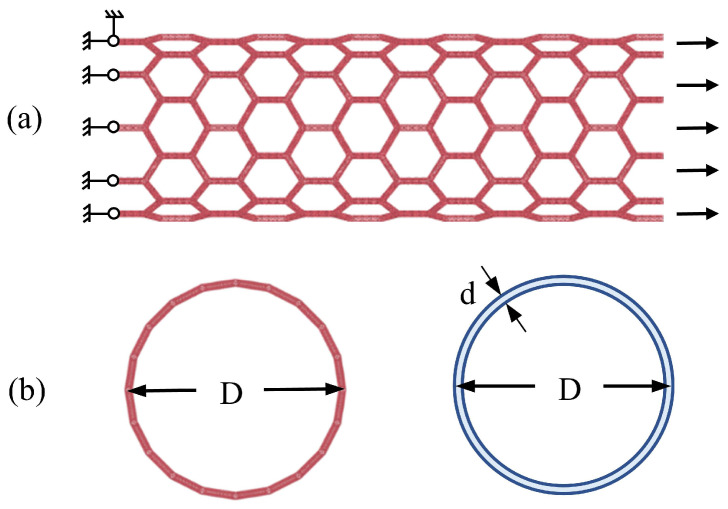
(**a**) Loading mode of the SCNT in MD simulations; (**b**) original and equivalent cross-section of the SCNT model (*D* and *d* are the diameters of SCNT and CNT, respectively).

All MD simulations were conducted via Large-scale Atomistic/Molecular Massively Parallel Simulator (LAMMPS) open-source code [47]. After the construction of the SCNT model, an energy minimization process was performed to obtain an equilibrium configuration with minimum potential energy. Subsequently, axial tension was applied through displacement loading, where one side moves and the other side remains constrained throughout the process(as shown in Figure 2a). Only the axial degree of freedom of two ends of SCNTs was restrained, allowing the SCNTs to deform freely in the radial direction. The temperature of the simulating system was fixed at 0.5 K to reduce thermal vibrations of atoms so that the analysis of detailed deformation of CNT units was more accessible. The Nosé–Hoover thermostat [48] was applied to maintain temperature stability. This temperature setting was validated as reasonable via comparisons with a previous study of SCNTs [49], which are presented in Figure S2 of the Supplementary Materials.

In this study, the loading rate was taken as 0.1 Angstrom/ps, which was determined based on previous MD simulations of CNT-based networks and a parametric study on graphene and CNT [50,51]. A recent study on the elastic straining of free-standing mono-layer graphene proved that the results of the experiments and MD simulations have reasonable agreements [52]. According to Newton’s third law, the resultant force in the tensile direction can be obtained by summing up the counter-force at the end of the SCNTs. The cross-section area of the SCNTs was approximated as a continuum of circular rings [49], as shown in Figure 2b. Accordingly, the cross-section stress of the SCNTs is calculated as the following formula:

(1) $$\sigma =\frac{F}{\pi Dd}$$

where *F* is the applied force, *D* is the diameter of the SCNT and *d* is the diameter of the CNT. The diameter of the SCNT is determined by the CNT length *l* and the chiral indices [*N*,*M*]. The diameter of the CNT is related to its chiral indices (*n*,*m*). Therefore, calculating the cross-section stress with this equation allows for the consideration of all geometry parameters in this study.

## 3. Results and Discussion

### 3.1. Influence of Geometrical Structure on Tensile Performances of SCNTs

**Figure 3:**
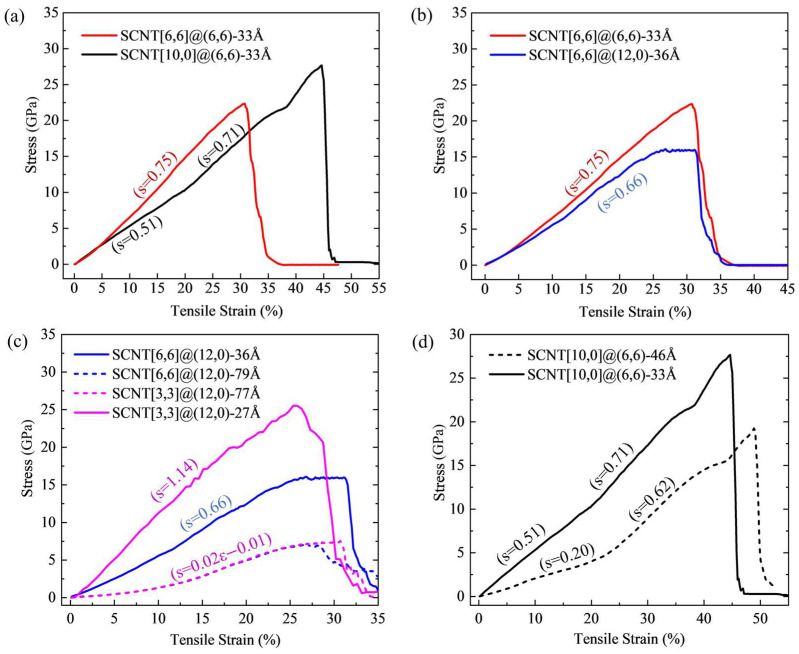
(**a**) Comparison of tensile curves for SCNTs with different CNT arrangements; (**b**) comparison of tensile curves for SCNTs with different CNT chirality; (**c**) comparison of tensile curves for armchair SCNTs with different CNT lengths; (**d**) comparison of tensile curves for zigzag SCNTs with different CNT lengths (S in parentheses indicates the curve slope obtained via the polynomial curve fitting).

Figure 3 presents the results of tensile curves for SCNTs with different geometrical structures. By conducting the polynomial curve fitting through the least square method, the slopes of all tensile curves were calculated. Each datum is located near the corresponding curve, which can be seen in Figure 3. The detailed curve fitting information can be found in Figure S3 of the Supplementary Materials.

In Figure 3a, the same CNT with a chirality of (6,6) and a length of 33Å is applied to fabricate two different SCNT structures: armchair SCNT[6,6]@(6,6) and zigzag SCNT[10,0]@(6,6). From Figure 3a, it is evident that these two SCNTs exhibit different tensile behaviors. The armchair SCNT[6,6]@(6,6) tends to have a linear stress–strain curve with a stable slope of 0.75. Differently, the tensile curve of the zigzag SCNT[10,0]@(6,6) has two linear stages with different curve slopes of 0.51 and 0.71.

In Figure 3b, the tensile curves of SCNT[6,6]@(6,6) and SCNT[6,6]@(12,0) are compared. They have the same network structure but are fabricated by CNTs with different chirality. The slope of the SCNT[6,6]@(12,0) is 0.66 on average before the strain of 0.2, which is slightly lower than that of the SCNT[6,6]@(6,6). This small difference is probably induced by the variation in CNT length. Hence, the two curves are quite similar before the stain of 20%. However, the difference between the two curves becomes remarkably larger after the strain of 25%. The curve of SCNT[6,6]@(6,6) remains linear, while the curve of SCNT[6,6]@(12,0) turns out to be a plateau in the later stage of tension. During this plateau, the deformation of SCNT[6,6]@(12,0) increases remarkably while the stresses remain almost unchanged, indicating ductile fracture characteristics. In contrast, SCNT[6,6]@(6,6) exhibits a typical brittle fracture performance according to the shape of its curve.

Figure 3c,d demonstrate the tensile curves of SCNTs with different CNT lengths, for armchair and zigzag SCNTs, respectively. In Figure 3c, two armchair SCNT structures are presented and each of them involves two models fabricated by CNTs with different lengths. According to the specific values of curve slopes, shorter CNTs apparently enable SCNTs to have a larger tangent elastic modulus for both two armchair SCNT structures. Correspondingly, the ultimate tensile stresses of the SCNTs with shorter CNTs are much larger than those of SCNTs with longer CNTs. Additionally, it is worth noting that the shapes of the tensile curves of the SCNTs change with the increase in CNT length. The curves of SCNT structures with shorter CNTs are approximately linear, but they become non-linear when the CNT lengths increase. Moreover, it is noted that the tensile curves of SCNT[6,6]@(12,0)-79Å and SCNT[3,3]@(12,0)-77Å overlap with each other during the tension. These structures have the same arrangement of CNT units but different sizes of diameter.

In Figure 3d, the tensile curves of SCNT[10,0]@(6,6) models with CNT unit lengths of 33Å and 46Å are compared. Both two SCNTs possess two-stage linear performances during the tension. In SCNT[10,0]@(6,6)-46Å, a noticeable turning point occurs at the strain of about 25%. The turning point of two stages of SCNT[10,0]@(6,6)-33Å is not explicit, but can still be identified according to the detailed slope data, which is at about the strain of 20%. Similarly to armchair SCNTs, zigzag SCNT[10,0]@(6,6) with shorter CNTs (length is 33Å) has higher tensile slopes in the early tension stage before the strain of 20%. However, with the increase in tensile strain, it is notable that the tensile slope of SCNT[10,0]@(6,6)-46Å in the second stage rises to more than 300% of the value in the first stage. Moreover, the slopes of these two curves are very close in the second linear stage, with the difference being less than 10%. This comparison indicates that the CNT length affects the tensile slopes of the zigzag SCNTs in the early strain stage. Figure 3c,d shows that the CNT length has a significant influence on the tangent elastic modulus for both the armchair and zigzag SCNTs. The underlying reason is related to the deformation mode of the SCNTs, which is discussed in the following section.

### 3.2. Deformation Mode Analysis for SCNTs with Different Geometrical Structures

From the previous discussion, conclusions can be obtained that the tensile performances of SCNTs are affected by the length, chirality and arrangement of CNTs. Furthermore, it should be noted that these geometrical parameters play independent roles at separate stages.

**Figure 4:**
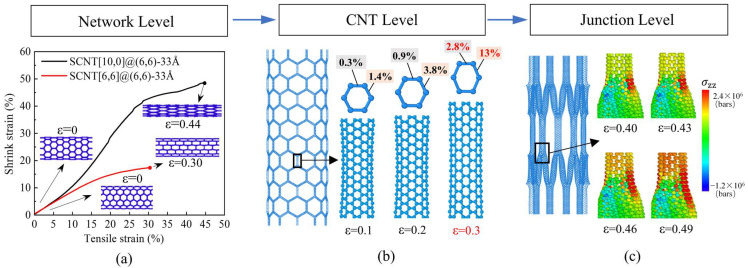
Hierarchical deformation process of SCNTs. (**a**) Network-level deformation of SCNT: remarkable shrinkage in the radial direction and different morphology transformations of armchair and zigzag SCNTs; (**b**) CNT-level deformation of SCNT: increments in CNT length and elongations of carbon bonds; (**c**) junction-level deformation of SCNT: expanding stress-concentration area of the junction.

This characteristic is essentially related to the distinctive hierarchical deformation mode of CNT-based networks, as illustrated in Figure 4. During tension, SCNTs undergo deformation at three levels: the network level, the CNT level and the junction level. In the early tensile stage, SCNT structures experience remarkable radial shrinkage primarily due to CNT rotation, which can be clearly seen in Figure 4a. This deformation happens at the network level, for which the morphology transformation differs between armchair and zigzag SCNTs. Accordingly, zigzag and armchair SCNTs show totally different ranges and tendencies of radial shrinkage. Similar deformation phenomena have been observed in [49] and [35], referred to as the aligning process of CNT units along the tensile direction and the fish-net behavior, respectively. An investigation using finite-element methods for CNT-based honeycomb structures reported considerable flexibility as well [37].

As the SCNT tension increases, deformation begins to happen at the CNT level. In Figure 4b, detailed topologies of the CNT under the strain states of 0.1, 0.2 and 0.3 are presented. Explicit overall elongations of the CNTs can be observed when the strain increases from 0.1 to 0.3, which is mainly contributed by the extensions of the carbon bonds. By analyzing the elongation data of the carbon bonds, it is further observed that the CNT bond length has a more remarkable increment when the strain rises from 0.2 to 0.3. This indicates a larger deformation of the CNT level during this tensile stage. A similar CNT-level deformation is also concluded in [49], while the analysis was based on the shape of the tensile curves without providing detailed deformation data.

As the stretching of SCNTs develops towards the final tensile stage before fracture, noticeable deformation occurs at the junction areas. Figure 4c presents the stress concentration states of the junction at four strain states, with an increment of 0.03 for each state. For the same strain increment, the stress concentration rises much more significantly at the local junction area when the strain increases from 0.46 to 0.49. As a result, the failures of the entire SCNTs originate from the junction area, where defects occur and cracks propagate. In the study of [35], the SCNT rupture is also observed to occur at regions near the SWCNT junctions.

**Figure 5:**
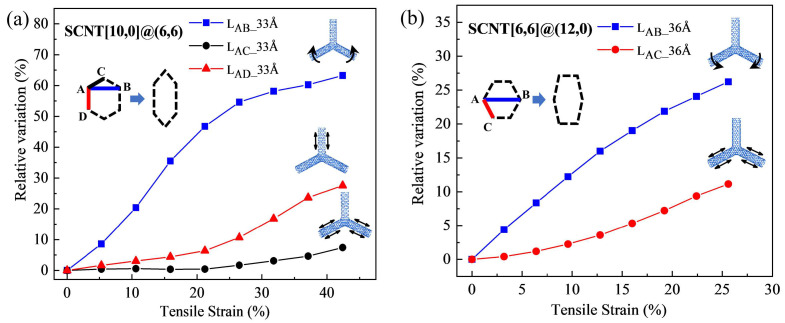
Morphology transformations for SCNTs with different CNT arrangements. (**a**) Zigzag SCNT[10,0]@(6,6); (**b**) armchair SCNT[6,6]@(12,0).

All SCNTs basically experience such hierarchical deformation processes during tension. However, different structures possess distinct features in detail, which is the essential reason for the varying tensile performances of SCNTs. To identify this issue, the morphology transformations of SCNTs were quantitatively evaluated with the increase of the tensile strains. Figure 5a and Figure 5b illustrate the morphology transformations for zigzag SCNT[10,0]@(6,6)-33Å and armchair SCNT[6,6]@(12,0)-36Å, respectively. The deformation of the SCNT structure can be represented by the deformation of hexagonal honeycomb units, which includes the CNT elongations and angle variations. Both the CNT elongation and the angle variation were measured by the length variation in the hexagonal honeycomb units. Relative variations were calculated for better comparisons.

First of all, it is evident that zigzag and armchair SCNTs have distinct deformation contributors in the hexagonal honeycomb unit. In the zigzag SCNT[10,0]@(6,6), length variations can be observed for both the vertical CNT (L_AD_) and the diagonal CNT (L_AC_), for which the elongation of the vertical CNT (L_AD_) is more dominant. In the honeycomb units of the armchair SCNT[6,6]@(12,0), there is only one type of CNT (L_AC_) that bears the elongation under tension. Apart from that, there are significant differences in the magnitude of deformation between zigzag and armchair SCNTs. CNTs have only 10% elongations in the armchair SCNT[6,6]@(12,0)-36Å, which is only one-third of their elongation in zigzag SCNT[10,0]@(6,6)-33Å. As for the angle rotation, zigzag SCNT[10,0]@(6,6)-33Å possesses an as large as 50% angle variation, but the data is only 25% for armchair SCNT[6,6]@(12,0)-36Å. These findings indicate that SCNTs with a zigzag structure tend to be more flexible than those with an armchair structure. Consequently, the zigzag SCNTs have a relatively lower tangent elastic modulus at the early stage and a larger range of tensile strain.

Furthermore, it should be noted that zigzag and armchair SCNTs exhibit different characteristics in the variation curves, indicating distinct deformation modes. In the case of the zigzag SCNT[10,0]@(6,6), the curve of the angle variation (blue line) initially has a considerable increase but gradually slows down after the strain of 20%. Conversely, the length variation curves (red and black lines) remain at a very low level at the early stage and start to increase after the strain of 20%. As for armchair SCNT[6,6]@(12,0), the increase in both the length and angle variation have almost stable slopes compared to those of zigzag SCNTs.

The differences in the deformation evolution curves uncover the distinct detailed deformation modes of zigzag and armchair SCNTs, which is the main reason for the different tensile performances demonstrated in the first part of the discussion. In zigzag SCNTs, the initial contributor to the tensile deformation is the angle variation, which gradually transitions into the elongation of CNTs after the strain of 20%. This is evident in the changing slopes and a turning point of the tensile curves of zigzag SCNT[10,0]@(6,6)-33Å at the strain of about 20%. Hence, the changing slopes of the tensile curves of zigzag SCNTs indicate a transformation in the deformation contributor throughout the entire tensile process. In zigzag SCNTs, the occurrences of two types of deformation are relatively independent, so they play separate roles during different tensile stages. On the other hand, for the armchair SCNT[6,6]@(12,0)-36Å, the morphology transformation in Figure 5b shows that two types of deformation occur simultaneously, resulting in a mixed mode that remains unchanged. For this reason, the slope of the tensile curve of the armchair SCNT[6,6]@(12,0)-36Å is stable and constant at the early stage. The linear tensile curves are induced by the simultaneous occurrence of the angle rotation and the CNT elongation.

**Figure 6:**
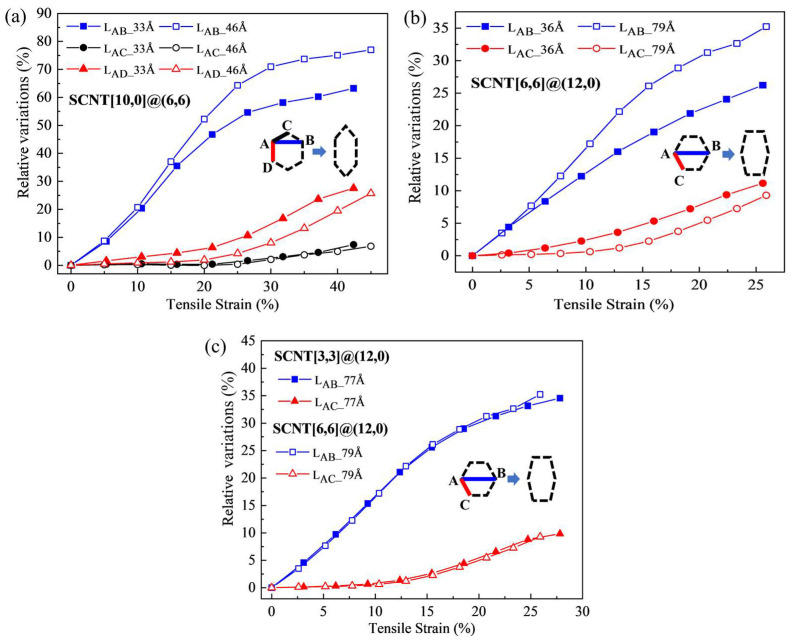
Morphology transformation for SCNTs with different CNT lengths. (**a**) Two zigzag SCNTs [10,0]@(6,6) with the CNT length of 33Å and 46Å; (**b**) two armchair SCNTs [6,6]@(12,0) with the CNT length of 36Å and 79Å; (**c**) two armchair SCNTs [3,3]@(12,0)-77Å and [6,6]@(12,0)-79Å.

The influence of the CNT length can also be reflected by the detailed morphology transformation of SCNTs. For both armchair and zigzag SCNTs, two models fabricated by different lengths of CNTs are compared, which are presented in Figure 6a and Figure 6b, respectively. Despite having different contributors of deformation, armchair and zigzag SCNTs possess similar changes when the CNT length increases. When longer CNTs are used, the starting points of CNT elongation (red curves) are prominently postponed for both armchair and zigzag SCNTs. In the zigzag SCNT[10,0]@(6,6), the starting point shifts from the strain of 15% to 25% with an increase in CNT length from 33Å to 46Å. In the armchair SCNT[6,6]@(12,0), the starting point transfers from 5% to 13% when CNT length increases from 36Å to 79Å. This postponement of the CNT elongation leads to a significant increase in angle variation deformation (blue lines) for both the armchair and zigzag SCNTs.

Such transformations of morphology evolution also indicate changes in the deformation mode of SCNTs, which are related to the variations of the tensile slopes. For both zigzag and armchair SCNTs, increasing the CNT length leads to the postponement of the CNT elongation during the tension. As a result, angle variation becomes the main contributor to the tension at the early stage, independent of the CNT elongation. The stiffness of angle variation is known to be much smaller than that of the CNT elongation. Therefore, the tensile slopes of both the armchair and zigzag SCNTs decrease when CNT lengths become longer.

Figure 6c provides a further comparison between SCNT[6,6]@(12,0)-79Å and SCNT[3,3]@(12,0)-77Å. In both models, the variation curves of all types of deformation coincide throughout the whole process. The tensile curves of these two SCNTs also completely overlap, as previously concluded. SCNT[6,6]@(12,0) and SCNT[3,3]@(12,0) are constructed by the approximate length and chirality of CNTs. Therefore, it can be concluded that CNT length plays a decisive role in controlling the deformation modes of SCNTs, compared to the effects of diameter and size.

To further analyze the effect of CNT length, the explicit relationship between the CNT length and the early tensile slope of the SCNT was identified based on the mechanics of materials. In the initial tensile process, the rotation deformation in SCNTs is approximately considered to involve only the bending of CNT units, regardless of the axial elongation. Under this assumption, the elastic modulus of SCNT was derived according to the deformation transformation and force balance of the Y-junction. The rotation deformation of the CNT unit is equivalent to the deflection of the cantilever beam. Details in deriving the equation are shown in Section S4 of the Supplementary Materials. Eventually, the relationship between the CNT length and the elastic modulus of SCNT is derived as

(2) $$E_{SCNT}=16\sqrt{3}\cdot \frac{E_{CNT}I_{CNT}}{d}\cdot \frac{1}{l^{3}}$$

where *d* and *l* are the diameter and the length of the CNT units and *E* and *I* are the elastic modulus and the cross-sectional moment of inertia of the SWCNT.

According to the expression of Equation (2), CNT length is the most prominent impact factor of *E_SCNT_*, since the *E_SCNT_* has a cubic inverse relationship with only the CNT length *l*. This equation directly explains the great importance of CNT length in the early stage of the tensile curves. However, this conclusion is more applicable to zigzag SCNTs than armchair SCNTs. This is because the CNT rotation continues to be an independent contributor to the tensile deformation of zigzag SCNT for a considerable period, which aligns with the hypothetical conditions of Equation (2). Zigzag SCNTs [10,0]@(6,6)-46Å and [10,0]@(6,6)-33Å can be taken as examples. The ratio of *E_SCNT_* between these two zigzag SCNTs is 0.35 based on Equation (2). In comparison, the ratio of the curve-fitting tensile slopes was calculated to be 0.39 for these two zigzag SCNTs, which is very close to the value of 0.35 obtained via theoretical derivation.

For armchair SCNT, CNT elongations cannot be ignored at the beginning of tension, so the applicability of Equation (2) is limited. Nevertheless, when CNT lengths are longer, the elongation is postponed so that armchair SCNTs are able to have a similar rotation-dominant deformation mode as zigzag SCNTs. Accordingly, Equation (2) is applicable for armchair SCNTs with longer CNT lengths. For instance, in this study, the ratio of *E_SCNT_* was calculated as 0.91 for armchair SCNTs [6,6]@(12,0)-79Å and [3,3]@(12,0)-77Å, which is in accordance with the overlapping performances of these two SCNTs in both tension curves and deformation transformation curves.

**Figure 7:**
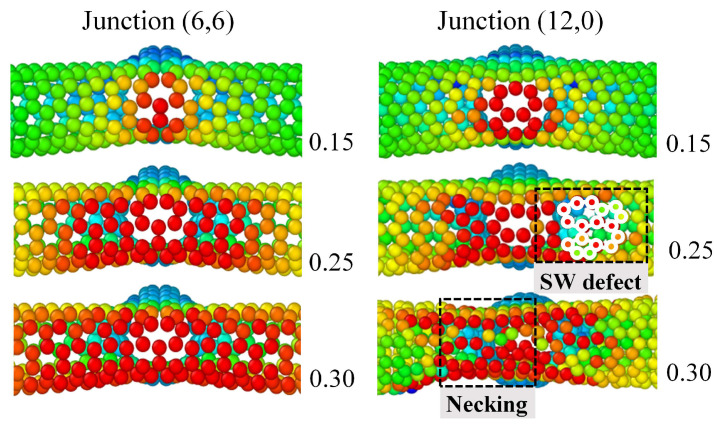
Topological details for the junction areas taken from SCNTs with different CNT chirality. Junctions are taken from the SCNT[6,6]@(6,6)-33Å and the SCNT[6,6]@(12,0)-36Å, respectively.

Regarding the CNT chirality, its impact becomes significant in the later stage of tensile curves, which was illustrated in the first part of the discussion. The corresponding reason is analyzed in Figure 7. There are two junction areas taken from the SCNT[6,6]@(6,6)-33Å and SCNT[6,6]@(12,0)-36Å, which have the same SCNT structure but are fabricated by CNTs with different chirality. Remarkable stress concentration can be seen for the junction areas of both two structures, but the two junctions present totally different topology structures. Under the strain of 0.25, distortions of hexagons are observed for the junction area connected by the (12,0) CNT, which is the so-called Stone–Wales defect as the precursor of fracture [53]. However, the junction topology structure of (6,6) CNT is unchanged under the same strain of 0.25. When the strains increase to 0.3, higher levels of stress concentration occur in the junction area. An explicit necking can be seen for the area of junction (12,0), while the atomic lattice of junction (6,6) is still unchanged. Therefore, the junction structure of (6,6) CNT is much more stable than that of the (12,0) CNT.

Corresponding to the tensile curves, the hexagonal topology distortion in (12,0) CNT induces the plateau of SCNT[6,6]@(12,0) as the plastic deformation. Differently, the stable topology of the (6,6) CNT makes the structure able to resist larger forces, resulting in a higher tensile strength. Therefore, it can be concluded that the topology stability of the junction area is significantly related to the chirality of CNTs and induces totally different fracture performance in the later tensile stages for CNT-based networks.

To summarize, the variable performances of SCNTs result from the transformation of deformation modes of the SCNTs and are affected by the CNT length, chirality and arrangements. The hierarchical nature of the deformation mode is the essential reason for the strain-dependent effect of geometry parameters on the tensile performances of SCNTs. In the early stage, deformation mainly happens on the network level, where the stiffness of the network structure is strongly influenced by the CNT length. This, in turn, affects the initial slope of the tensile curve of SCNTs. Gradually, the deformation is transferred into the CNT level and local junction level, where the topological chirality of CNT is more decisive. Therefore, the later stage and the fracture mode of the SCNT vitally depend on the CNT chirality. As for the CNT arrangement, the overall deformation pattern of the SCNT is fundamentally related to the CNT arrangement, which can explain why the CNT arrangement determines the overall shapes of tensile curves for SCNTs.

### 3.3. Summary of the Structure-Dependent Performances of SCNTs and the Potential Design Strategy

Based on previous discussions, it can be summarized that the mechanical properties of SCNTs are controllable and adjustable. The tensile curve shapes, the early tangent elastic modulus and the fracture performances of the SCNTs change remarkably with the variation of geometrical parameters including CNT arrangement, CNT length and CNT chirality.

Moreover, these geometrical parameters of SCNT structure play independent roles at separate stages of tension, which is an attractive feature for structural design. This feature is essentially induced by the hierarchical deformation mode of SCNT. The deformation of the SCNT progresses from the network level to the CNT-unit level and eventually to the junction level. Each level of deformation corresponds to a specific geometry parameter.

**Figure 8:**
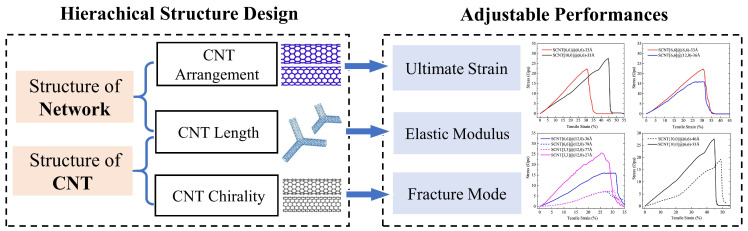
The design strategy of SCNTs based on the hierarchical structure.

With a comprehensive understanding of the structure-dependent mechanical performances of SCNTs, a basic design strategy based on the hierarchical structure can be developed for SCNTs (illustrated in Figure 8). The major factors influencing the geometrical parameters, such as CNT arrangement, CNT length and CNT chirality, can be divided into two levels of structure: the structure of the CNT and the structure of the SCNT. Accordingly, changes in structure at both the CNT and network levels significantly affect the mechanical performance of the resulting models. It is worth noting that the CNT length belongs to both the group of network structure and CNT structure. Therefore, the CNT length plays multiple roles in the mechanical performances of SCNTs. It was concluded by previous discussions that CNT length determines the starting point of CNT stretching and affects the ranges of the angle variation at the same time.

Apart from the SCNT, the obtained strategy of structural design can also be valuable for many other CNT-based structures, such as super graphene and pillared graphene [33,54]. Fabricated using CNTs or other building blocks, many CNT-based structures have similar hierarchical deformation processes as SCNTs. These structures also undergo the deformation transformation from the structure level to the junction level, so they are affected by similar geometrical parameters. Furthermore, the same principle of the hierarchical deformation process may also lead to respective stages for the geometry parameters of other CNT-based networks.

Moreover, this study’s findings are also useful for the practical application of hierarchical honeycomb structures. In experimental studies, similar characteristics can be found for the hierarchical honeycomb structure. For instance, multi-stage curves with varying stiffness are observed for the compression performance of a self-similar honeycomb structure, which is related to the hierarchical deformation [55]. A study of a hierarchical re-entrant honeycomb structure [56] found competition between the different levels of deformation, in agreement with the hierarchical deformation mode identified in this study. In the experimental investigations of honeycomb structures, the relative density, wall thickness or side length of the structures are usually found to be the major impact factors of the elastic modulus of the resulting materials [57,58], which can be optimized via the theoretical conclusions.

Via a reasonable structural design, a variety of adjustments can be realized for CNT-based networks, leading to more targeted and effective applications. Supported by the rapid development of technology, it is believed that performance-oriented designs can be achieved for CNT-based structures in the near future and be able to guide practical applications.

## 4. Conclusions

Through MD simulations, the influence of geometrical structure on the mechanical performances of the SCNT was investigated and the underlying mechanism was revealed by analyzing the structure evolution. The following conclusions may be drawn from the discussions.

Firstly, the tensile performance of the SCNT is structure-dependent, varying with changes in geometrical parameters including CNT arrangement, CNT length and CNT chirality. The CNT arrangement shapes the overall tensile curves of SCNTs, while the CNT length crucially affects the tangent elastic modulus of SCNTs at the early stage. CNT chirality plays an important role in the fracture process of SCNTs at the final stage.

Secondly, the influence of the geometrical structure is related to the deformation mode of SCNTs. All SCNTs undergo a hierarchical deformation process transferring from the angle variation at the network level to the CNT elongation and finally into the bond stretching at the local junction level. Altering the geometrical structure induces variations in the deformation mode of the SCNTs, resulting in different mechanical performances of SCNTs. Different CNT arrangements lead to different ranges of angle variation and different deformation contributors for armchair and zigzag SCNTs, creating different tensile flexibility and overall tensile curves of SCNTs. Increasing the CNT length can postpone the beginning of the CNT-stretching deformation and enlarges angle variation deformation, decreasing the tangent elastic modulus of all SCNTs at the early stage. CNT chirality affects the fracture mode of SCNTs because it determines the stability of the atomic topology at the junction area, which is the commencement of crack propagation.

Thirdly, a design strategy is developed for SCNTs based on the influence of geometry parameters. A performance-oriented design can be achieved for desired properties and different functions and may serve as a reference for other CNT-based networks or hierarchical honeycomb structures since hierarchical deformation is a common principle for all similar structures.

A comprehensive understanding of the influence of geometrical structures can provide valuable guidance for the structural design of CNT-based networks. With proper structural design, it is believed that the applications of CNT-based networks can be greatly optimized and more targeted.

## Figures and Tables

**Table 1:** Geometry parameters of SCNT structures in this study.

SCNT Models	CNT			SCNT		
	Diameter (Å)	Length (Å)	Aspect Ratio	Diameter (Å)	Length (Å)	Aspect Ratio
[10,0]@(6,6)-46Å	8.14	46.36	5.70	255.60	759.06	2.97
[10,0]@(6,6)-33Å	8.14	32.65	4.01	180.01	526.46	2.92
[6,6]@(6,6)-33Å	8.14	33.06	4.06	189.42	597.31	3.15
[6,6]@(12,0)-36Å	9.40	36.13	3.84	207.01	624.64	3.02
[6,6]@(12,0)-79Å	9.40	79.28	8.43	454.24	1156.76	2.55
[3,3]@(12,0)-77Å	9.40	76.79	8.17	219.99	646.85	2.94
[3,3]@(12,0)-27Å	9.40	26.71	2.84	76.52	205.26	2.68

## Data Availability

The data presented in this study are available on request from the corresponding author.

## Supplementary Materials

The following supporting information can be downloaded at: https://www.mdpi.com/article/10.3390/nano13243119/s1, Figure S1: (a) The unrolled honeycomb lattice of SWCNT for geometry illustration; (b) Construction process of SCNT and the geometry illustration; Figure S2: Comparisons of tensile curves between results in reference [49] and in this study. Figure S3: Curve fittings for tensile curves of different SCNTs in this study; Figure S4: Deformation and force analysis of Y-junction in zigzag SCNTs; Figure S5: Deformation and force analysis of Y-junction in armchair SCNTs.
