# Supplementary material for: Understanding the Mechanism of the Structure-Dependent Mechanical Performance of Carbon-Nanotube-Based Hierarchical Networks from a Deformation Mode Perspective
Source: Nanomaterials (Basel). 2023 Dec 12;13(24):3119. doi: 10.3390/nano13243119 (PMC10745568; doi:10.3390/nano13243119)
Supplement: Supplementary file 1 [file nanomaterials-13-03119-s001.zip › nanomaterials-2704364-supplementary.pdf]

S1. Detailed illustration of the geometry structure of the CNT and the SCNT

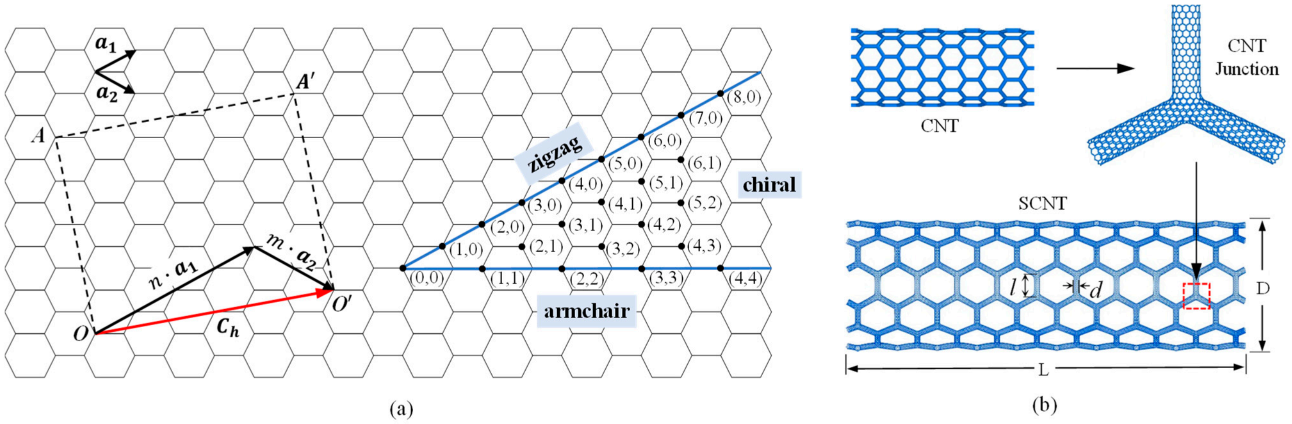

Figure S1 (a) The unrolled honeycomb lattice of SWCNT for geometry illustration; (b) Construction process of SCNT and the geometry illustration.

The SWCNT is rolled up from the single-layered graphene. As illustrated in Fig. S1(a), the structure of a single-walled carbon nanotube is characterized by several key parameters. The chiral vector $\boldsymbol{C_h}$ connects two equivalent sites in a graphene lattice, which are expressed in terms of the unit vectors $\boldsymbol{a_1}$ and $\boldsymbol{a_2}$. The chiral vector $\boldsymbol{C_h}$ corresponds to the direction perpendicular to the nanotube axis and can be expressed as

$$\boldsymbol{C_h} = n\boldsymbol{a_1} + m\boldsymbol{a_2} \equiv (n, m) \tag{S1}$$

where n and m are integers and they are commonly in pairs to be called chiral indices (n, m). Chiral Angle θ is the angle between the chiral vector $\boldsymbol{C_h}$ and the vector $\boldsymbol{a_1}$. It ranges from 0 to 30 degrees. Nanotube symmetry groups are classified as the chiral angle, including zigzag (if $\theta = 0°$), armchair (if $\theta = 30°$), or chiral ($0° < \theta < 30°$). Based on the chiral vector $\boldsymbol{C_h}$, the diameter of the SWCNT is calculated as

$$d = \frac{|\boldsymbol{C_h}|}{\pi} = \frac{\sqrt{3}a}{\pi}\sqrt{n^2 + m^2 + nm} \tag{S2}$$

where a is the lattice constant of the graphene sheet (approximately 0.246 nm), and n and m are the chiral indices.

The SCNT has a similar geometry as the SWCNT. To construct an SCNT, SWCNTs are connected into Y-shaped junctions, and then these junctions are assembled into the resulting structures, as illustrated in Fig. S1(b). L and D represent the length and diameter of the SCNT, while l and d are the length and the diameter of the CNT. Since the SCNT follows the same geometry principle as the SWCNT, its structure is defined by similar parameters, including the chiral vector and the nanotube symmetry groups. The chiral indices of the SCNT are expressed as [N, M] to differentiate them from those of the CNT. The diameter of an SCNT is given by

$$D = \frac{l}{\pi}\sqrt{3(\mathrm{N}^2 + \mathrm{M}^2 + \mathrm{NM})} \tag{S3}$$

where l is the length of SWCNT and N and M is the chiral indices of the SCNT.

S2. Validation of MD simulations under 0.5K in this study

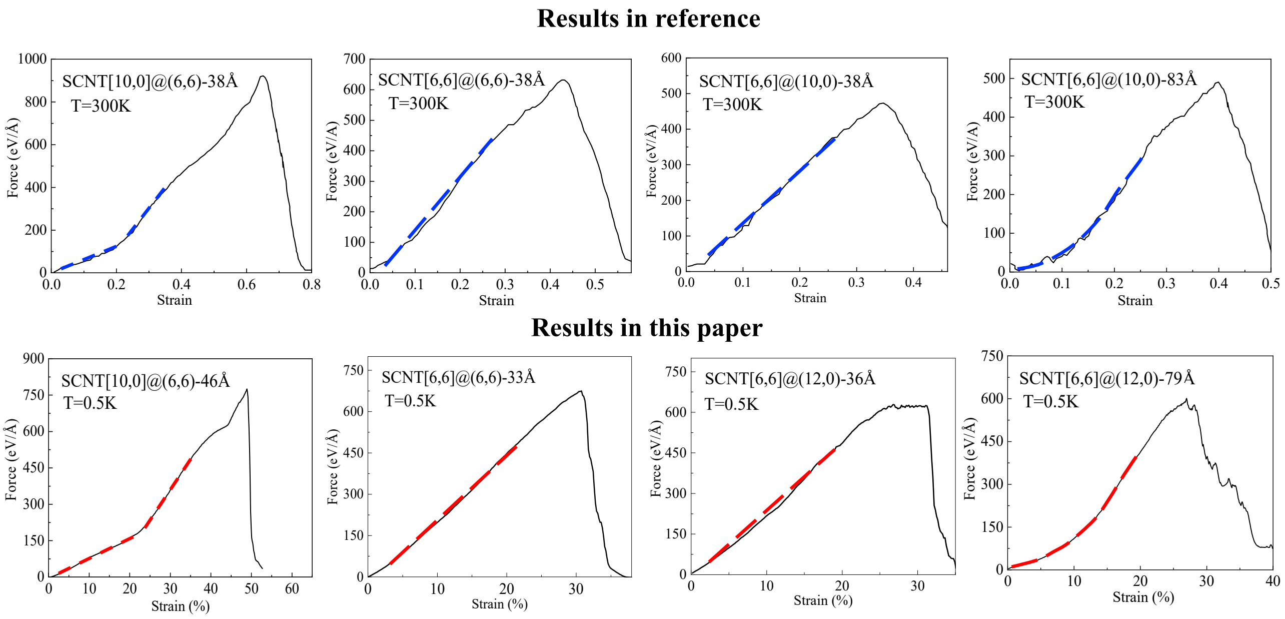

Figure S2 Comparisons of tensile curves between results in reference [49] and in this study.

Tensile curves obtained in this study (0.5K) are compared with the curves in [49] (300K), which are presented in Fig. S2. There are four groups of comparisons for SCNTs. In each group, the SCNTs taken from the reference [49] have close geometrical parameters to the SCNTs in this study (same types of chirality at both the CNT and SCNT level, approximate CNT length).

First of all, the tensile curves of SCNTs in this study (0.5K) have very similar shapes to the corresponding curves of the same types of SCNTs at 300K in Ref [49]. For each group, such similarities in the characteristics of the tensile curves are stable for a considerable period, covering the early and middle stages of the entire tension process of SCNTs.

Furthermore, the essential rules and conclusions on the structure-dependent tensile curves of SCNT do not change as the vary of temperature settings. The influencing rule of the CNT length remains the same: increasing the CNT length leads to a reduction in the initial slopes of the curves. These similarities exist for both the armchair and zigzag SCNTs.

S3. Curve fittings for the tensile curves of the SCNTs

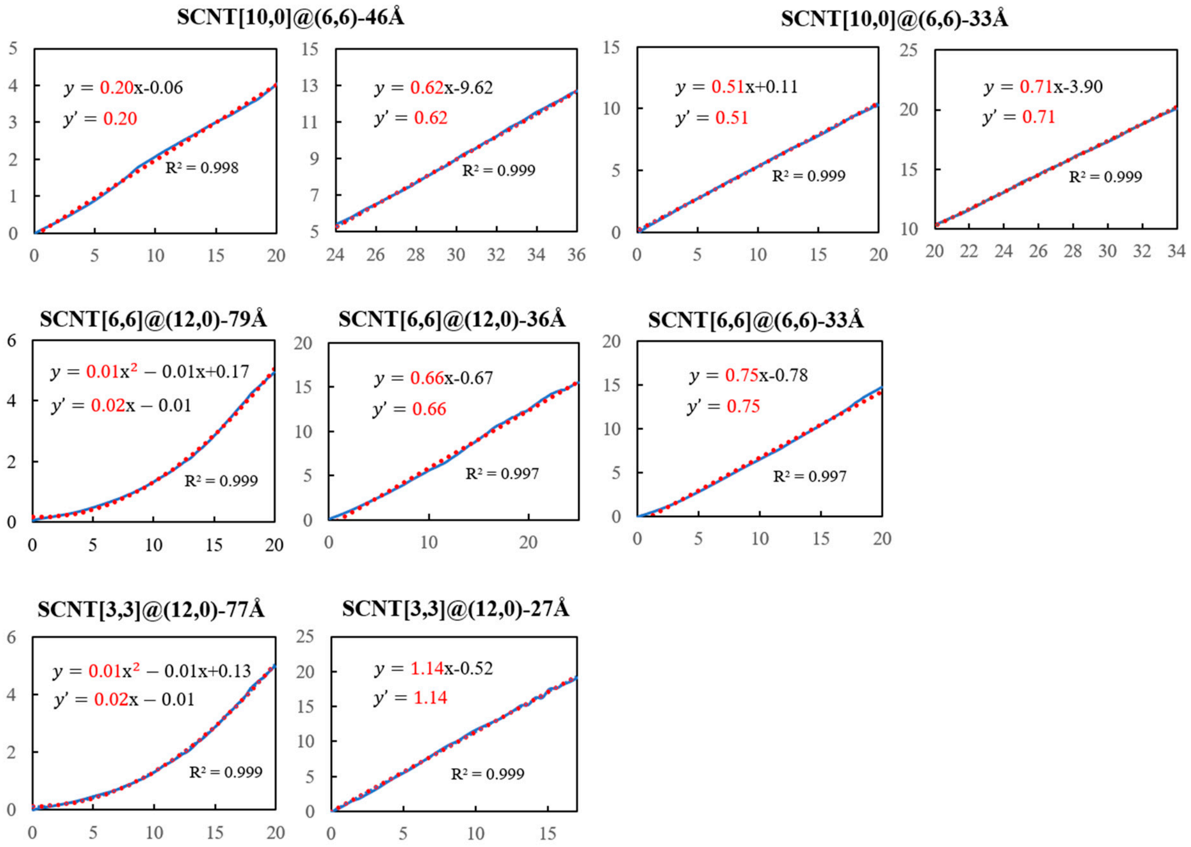

Figure S3 Curve fittings for tensile curves of different SCNTs in this study.

Polynomial curve fitting is conducted based on the least square method. Due to the different morphological characteristics of SCNT curves, various polynomials are utilized for curve fittings. Curves with overall linear shapes (curves for SCNT[6,6]@(12,0)-36Å, SCNT[6,6]@(6,6)-33Å, SCNT[3,3]@(12,0)-27Å) are fitted with the simplest unitary primary polynomial functions. Nonlinear curves (SCNT[6,6]@(12,0)-79Å, SCNT[3,3]@(12,0)-77Å) are fitted with the quadratic polynomial functions in one variable. For curves that are linear in multi-stage (SCNT[10,0]@(6,6)-46Å and SCNT[10,0]@(6,6)-33Å), the fittings are performed in corresponding stages. Considering the unstable fluctuations of data in the later stage of tension or the turning stage of the multi-stage curves, data used for curve fitting are taken from the more stable ranges. The results are presented in Fig. S3. The goodness of fit statistics can be identified according to the R-squared values, for which it can be seen that the curve fitting is justified. The obtained fitting trend lines agree well with the original curves. By deriving the resulting fitting functions, the curve slopes of SCNTs can be determined.

S4. Details in deriving the E_{SCNT}

For both the zigzag and armchair SCNTs, the minimum repeatable unit, Y-junctions, is taken to analyze the deformation transformation and force balance, which are illustrated in Fig. S4 and Fig. S5 for zigzag and armchair SCNTs, respectively. The strain of the Y-junction can be derived based on the 2D geometric relationships. The length of the SWCNT is denoted as l, and each branch of the Y-junction has a length of $l/2$. The initial state of the junction is colored as gray and the junction at the state after the deformation is blue.

S4.1 Zigzag SCNTs

For zigzag SCNTs, the CNT units of the Y-junction experience inwards rotation under Z-axial tensile forces, as shown in Fig. S4. The initial length a_0 of the Y-junction body is calculated to be $0.75l$ along the Z axis from Eq. (S5). In the stretched body of the Y-junction, the initial length a_0 becomes a, and there exists $a = a_0 + \Delta a$. The deformation Δa equals to the length of the projection of w onto the Z-axis with the expression in Eq. (S6). With the assumption of CNT bending, w is approximately the deflection of the cantilever rod under the vertical action of the concentrated force P at the rod end, which is calculated as Eq. (S7). E and I are the elastic modulus and cross-sectional moment of inertia of the SWCNT, for which the cape marking is applied to specifically refer to the parameter of SWCNT. The strain of the Y-junction can be obtained based on Eq. (S4), where z is the number of Y-junction units along the Z-direction of the SCNT model. The result is presented as Eq. (S8).

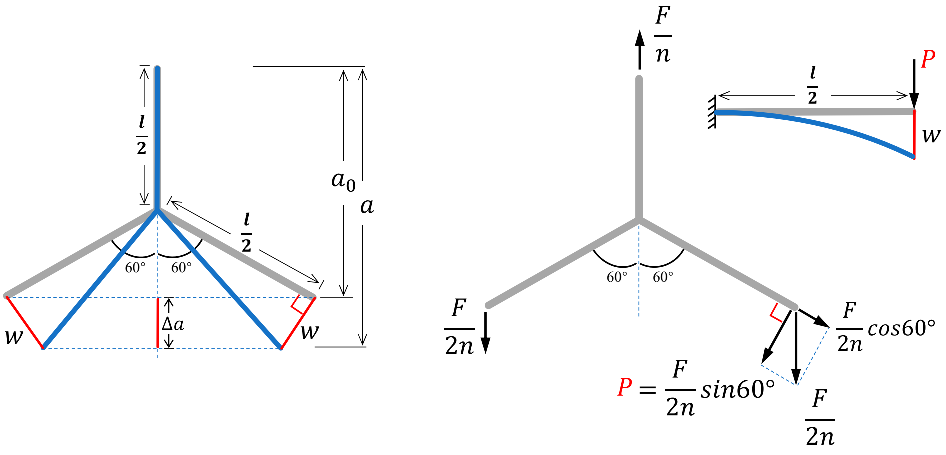

Figure S4 Deformation and force analysis of Y-junction in zigzag SCNTs.

The deflection w is induced by the force P. Based on the force balance, the relationship between the inducing force P and the total tensile force F can be identified. F acts on the entire SCNT model along the Z-direction, so the internal force in the top branch of the Y-junction unit is F/n, where n is the number of the Y-junctions along the radial direction of SCNTs. For the lower two branches of the Y-junction, their internal forces are half of that of the top one (i.e. $F/2n$) due to the force balance in the Z-axis. The direction of force P should be vertical to the direction of the bar, so the force P is the projection of the internal forces $F/2n$ in the vertical direction. With the projection geometry relationship, the tensile force F can be expressed as the function of P as in Eq. (S10). SCNTs are regarded as continuum circular rings to calculate the cross-section stress and the stress calculation follows Eq. (S9), where D and d represent the diameters of the SCNT and SWCNT, respectively.

$$\varepsilon = \frac{\Delta L}{L_0} = \frac{z \cdot \Delta a}{z \cdot a_0} = \frac{\Delta a}{a_0} \tag{S4}$$

$$a_0 = \frac{l}{2} + \frac{l}{2} \cdot cos60° = \frac{3}{4} l \tag{S5}$$

$$\Delta a = sin60° \cdot w = \frac{\sqrt{3}}{2} w \tag{S6}$$

$$w = \frac{P\left(\frac{l}{2}\right)^3}{3E_{CNT}I_{CNT}} \tag{S7}$$

$$\varepsilon = \frac{\Delta a}{a_0} = \frac{\frac{\sqrt{3}}{2}\cdot\frac{P\left(\frac{l}{2}\right)^3}{3E_{CNT}I_{CNT}}}{\frac{3}{4}l} = \frac{\sqrt{3}\cdot Pl^2}{36\cdot E_{CNT}I_{CNT}} \tag{S8}$$

$$\sigma = \frac{F}{A} = \frac{F}{\pi Dd} \tag{S9}$$

$$F = \frac{2n\cdot P}{sin60°} = \frac{4nP}{\sqrt{3}} \tag{S10}$$

$$E_{SCNT} = \frac{\sigma}{\varepsilon} = \frac{\frac{\frac{4nP}{\sqrt{3}}}{\pi Dd}}{\frac{\sqrt{3}\cdot Pl^2}{36\cdot E_{CNT}I_{CNT}}} = \frac{48}{\pi}\cdot\frac{E_{CNT}I_{CNT}}{d}\cdot\frac{n}{l^2\cdot D} \tag{S11}$$

$$n = N + M = N \quad (M = 0) \tag{S12}$$

$$D = \frac{l\cdot\sqrt{3(N^2+NM+M^2)}}{\pi} = \frac{\sqrt{3}N\cdot l}{\pi} \tag{S13}$$

$$E_{SCNT} = \frac{48}{\pi}\cdot\frac{E_{CNT}I_{CNT}}{d}\cdot\frac{N}{l^2\cdot\frac{\sqrt{3}N\cdot l}{\pi}} = 16\sqrt{3}\cdot\frac{E_{CNT}I_{CNT}}{d}\cdot\frac{1}{l^3} \tag{S14}$$

With the obtained equations of strain and stress, the E_{SCNT} of the zigzag SCNT is initially derived as Eq. (S11). D and n can be expressed with the parameters of the SCNT chirality vector *(N, M)* and SWCNT length l, which are presented in Eqs. (S12) and (S13). Substituting Eq. (S12) and (S13) into Eq. (S11), the expression of E_{SCNT} can be finally simplified as the Eq. (S14).

The ratio of E_{SCNT} for zigzag SCNTs [10,0]@(6,6)-46Å and [10,0]@(6,6)-33Å are calculated with Eq. (S14). Due to the same chirality settings of both SCNT and SWCNT, the items of d, E_{CNT} and I_{CNT} are the same. The corresponding expression can be simplified as follows. Subscripts use the value of CNT length to distinguish two SCNT models. The data for CNT length and SCNT diameter are taken from Table 1 in the main body of this paper.

$$\frac{E_{S-46}}{E_{S-33}} = \frac{(l_{33})^3}{(l_{46})^3} = \frac{(32.65)^3}{(46.36)^3} = 0.35$$

S4.2 Armchair SCNTs

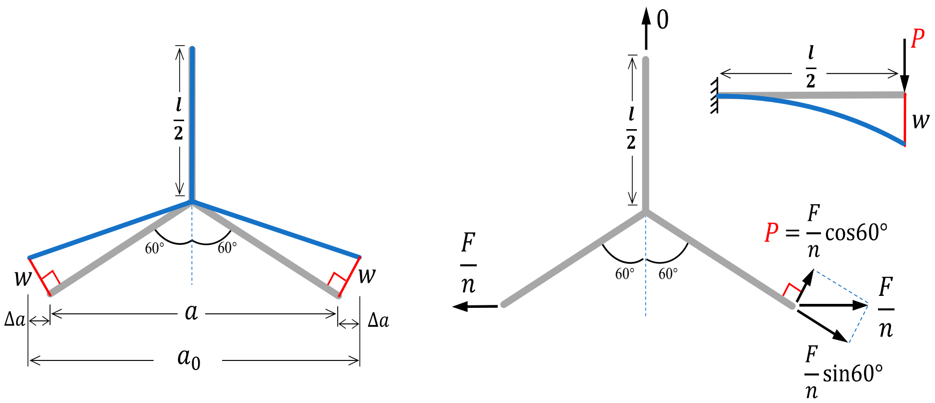

Figure S5 Deformation and force analysis of Y-junction in armchair SCNTs.

For armchair SCNTs, the derivation process of E_{SCNT} is similar to that of the zigzag SCNT. Yet there are still some differences in detail. For armchair SCNTs, the CNT units of Y-junction experience outwards rotation under the Y-axial tensile forces, which are presented in Fig. S5. Accordingly, the lengths of a_0 and Δa change (shown in Eqs. (S16) and (S17)) and there exists $a = a_0 + 2\Delta a$ for the stretched body of Y-junction in armchair SCNTs. Besides, the value and direction of the internal force are different,

as the illustration in Fig. S5. In armchair SCNTs, the tensile force is applied along the horizontal direction of the Y-junction and bared by only the two lower branches. As a result, the internal force of each lower branch is twice that of zigzag SCNTs and the horizontal loading direction leads to different triangular function relations between P and F, which are presented in Eq. (S21). E_{SCNT} is derived with the same approach and a similar expression of Eq. (S22) is obtained, in which the constant changes and the rest remains unchanged. Similar to the zigzag SCNT, the D and n of armchair SCNT are simplified as the SCNT chirality vector and the CNT length. The final expression of the E_{SCNT} is presented in Eq. (S25). Details of derivations are presented as follows.

$$\varepsilon = \frac{\Delta L}{L_0} = \frac{y \cdot 2 \cdot \Delta a}{y \cdot a_0} = \frac{2\Delta a}{a_0} \tag{S15}$$

$$a_0 = 2 \cdot \frac{l}{2} \cdot sin60° = \frac{\sqrt{3}}{2} l \tag{S16}$$

$$\Delta a = cos60° \cdot w = \frac{1}{2} w \tag{S17}$$

$$w = \frac{P\left(\frac{l}{2}\right)^3}{3E_{CNT}I_{CNT}} \tag{S18}$$

$$\varepsilon = \frac{2\Delta a}{a_0} = \frac{\frac{P\left(\frac{l}{2}\right)^3}{3E_{CNT}I_{CNT}}}{\frac{\sqrt{3}}{2} l} = \frac{\sqrt{3} \cdot Pl^2}{36E_{CNT}I_{CNT}} \tag{S19}$$

$$\sigma = \frac{F}{A} = \frac{F}{\pi D d} \tag{S20}$$

$$F = \frac{n \cdot P}{cos60°} = 2np \tag{S21}$$

$$E_{SCNT} = \frac{\sigma}{\varepsilon} = \frac{\frac{2nP}{\pi D d}}{\frac{\sqrt{3} \cdot Pl^2}{36E_{CNT}I_{CNT}}} = \frac{24\sqrt{3}}{\pi} \cdot \frac{E_{CNT}I_{CNT}}{d} \cdot \frac{n}{l^2 \cdot D} \tag{S22}$$

$$n = N + M = 2N \quad (N = M) \tag{S23}$$

$$D_{SCNT} = \frac{l \cdot \sqrt{3(N^2 + NM + M^2)}}{\pi} = \frac{3N \cdot l}{\pi} \tag{S24}$$

$$E_{SCNT} = \frac{24\sqrt{3}}{\pi} \cdot \frac{E_{CNT}I_{CNT}}{d} \cdot \frac{2N}{l^2 \cdot \frac{3N \cdot l}{\pi}} = 16\sqrt{3} \cdot \frac{E_{CNT}I_{CNT}}{d} \cdot \frac{1}{l^3} \tag{S25}$$

The ratio of E_{SCNT} for armchair SCNTs [6,6]@(12,0)-79Å and [3,3]@(12,0)-77Å is calculated with Eq. (S25). These two SCNTs are fabricated by the CNTs with same chirality and length, so the items of d, E_{CNT} and I_{CNT} are the same. The corresponding expression is simplified as follows. Subscripts use the chirality vector at the SCNT level to distinguish two SCNT models. The data for CNT length and SCNT diameter are taken from Table 1 in the main body of this paper.

$$\frac{E_{S-[6,6]}}{E_{S-[3,3]}} = \frac{(l_{77})^3}{(l_{79})^3} = \frac{(76.79)^3}{(79.28)^3} = 0.91$$
